# Circular RNAs and Drug Resistance in Genitourinary Cancers: A Literature Review

**DOI:** 10.3390/cancers14040866

**Published:** 2022-02-09

**Authors:** Gongwei Long, Siquan Ma, Runlin Shi, Yi Sun, Zhiquan Hu, Ke Chen

**Affiliations:** 1 Department of Urology, Tongji Hospital, Tongji Medical College, Huazhong University of Science and Technology, Wuhan 430030, China; longgw@hust.edu.cn (G.L.); M202076290@hust.edu.cn (S.M.); yisun21@tjh.tjmu.edu.cn (Y.S.); 2 Hubei Institute of Urology, Tongji Hospital, Tongji Medical College, Huazhong University of Science and Technology, Wuhan 430030, China; 3 Department of Urology, The Second Affiliated Hospital of Nanchang University, Nanchang 330006, China; ndefy19437@ncu.edu.cn

**Keywords:** circular RNA, drug resistance, renal cell carcinoma, bladder cancer, prostate cancer

## Abstract

**Simple Summary:**

Drug resistance to systematic treatment in genitourinary tumors severely aggravated the burden on patients and society. Multiple mechanisms were involved in drug resistance. As typical non-coding RNAs, circRNAs play a critical role in the onset and development of cancers and several studies implied their function in the regulation of drug resistance. Here, we reviewed the investigations of circRNAs’ behavior in drug resistance of genitourinary cancers and summarized the underlying mechanisms. This review emphasized the essential role of circRNAs in drug resistance development and also pointed out the potential topics that need further investigations in the future.

**Abstract:**

In recent years, systematic treatment has made great progress in genitourinary tumors. However, some patients develop resistance to the treatments, resulting in an increase in mortality. Circular RNAs (circRNAs) form a class of non-coding RNAs with high stability and significant clinical relevance. Accumulating evidence indicates that circRNAs play a vital role in cancer development and tumor chemotherapy resistance. This review summarizes the molecular and cellular mechanisms of drug resistance mediated by circRNAs to common drugs used in the treatment of genitourinary tumors. Several circRNAs were identified to regulate the responsiveness to systemic treatments in genitourinary tumors, including chemotherapies such as cisplatin and targeted therapies such as enzalutamide. Canonically, cicrRNAs participate in the competing endogenous RNA (ceRNA) network, or in some cases directly interact with proteins, regulate downstream pathways, and even some circRNAs have the potential to produce proteins or polypeptides. Several cellular mechanisms were involved in circRNA-dependent drug resistance, including autophagy, cancer stem cells, epithelial-mesenchymal transition, and exosomes. The potential clinical prospect of circRNAs in regulating tumor drug resistance was also discussed.

## 1. Introduction

Genitourinary cancers, including renal cell carcinoma (RCC), bladder cancer (BCa), prostate cancer (PCa), and other less common cancers, were estimated to have 362,860 newly diagnosed cases and lead to 68,030 deaths in the United States in 2020 [1]; especially since PCa has the greatest number of new cases and the 2nd greatest number of deaths in men, and BCa ranked 4th in new cases and 8th in deaths in men. Radical treatments with curative purposes were recommended for early-stage cancers, such as radical and partial nephrectomy for RCC [2], transurethral resection of bladder tumors and radical cystectomy for BCa [3,4], as well as radical prostatectomy (RP) and radical radiation therapy (RT) for PCa [5], but when the cancers progressed to advanced or even metastatic stages, the cancers are no longer curable by surgical treatments and systemic therapy would be the primary choice that could effectively extend survival [6,7,8]. Nevertheless, systemic therapy resistance is inevitable for some patients, which is a serious obstacle in cancer management, and behind it is a complex process involving multiple mechanisms.

**Figure 1:**
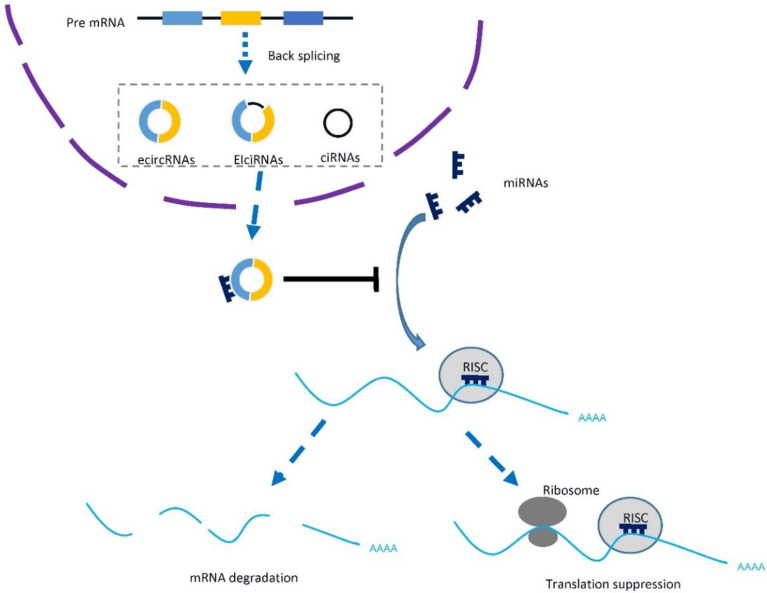
Biogenesis and function of circRNAs. CircRNAs were generated by back splicing of precursor mRNAs, and they can be classified as exonic circRNAs (ecircRNAs), exon-intron circRNAs (EIciRNAs), and circular intronic RNAs (ciRNAs) based on the introns or exons it contains. Typically, circRNAs sponge micro RNAs and rescue their repression of downstream mRNAs. RISC: RNA-induced silencing complex.

Circular RNA (circRNA) is a typical class of non-coding RNAs that were produced in the back-splicing process between the 5′ splice donor and 3′ splice acceptor site of precursor messenger RNA (mRNA), which forms a closed circular structure (Figure 1) [9]. Due to different splicing processes, three main types of circRNAs were generated, including exonic circRNAs (ecircRNAs), exon-intron circRNAs (EIciRNAs), and circular intronic RNAs (ciRNAs) [10]. The back-splicing is less efficient than canonical splicing, therefore both *cis*-elements and *trans*-factors are required. Studies have reported that the circRNAs generation can be facilitated by either RNA pairing of reversely complementary sequences across their flanking introns [11] or proteins binding to pre-mRNAs to bridge flanking introns together [12]. In cancers, the more frequent chromosomal translocations induce the formation of cancer-specific fusion circRNAs [13], while aberrantly expressed splicing factor also regulates the biogenesis of circRNAs in cancer [14].

With the rapid development of high-throughput sequencing technologies and novel approaches of functional characterization, the identification and relevant mechanisms of circRNAs have been widely explored [15]. RNA-sequencing (with or without RNase R) could identify novel circRNAs and well-designed microarrays, which are also efficient tools for circRNA profiling [16]. For already known circRNAs, experimental approaches, including PCR [17], northern blotting [18], and RNA fluorescence in situ hybridization (FISH) [19], are feasible for the validation and quantification of circRNAs. The best-studied mechanism of circRNA function is the competing endogenous RNA (ceRNA) network, in which circRNAs sponge micro RNAs (miRNAs) and regulate the downstream messenger RNA (mRNA) level (Figure 1). Other mechanisms, including interacting with RNA binding proteins (RBPs) [20], regulating transcription or splicing [12,19], and translating proteins [21], were also increasingly studied.

**Figure 2:**
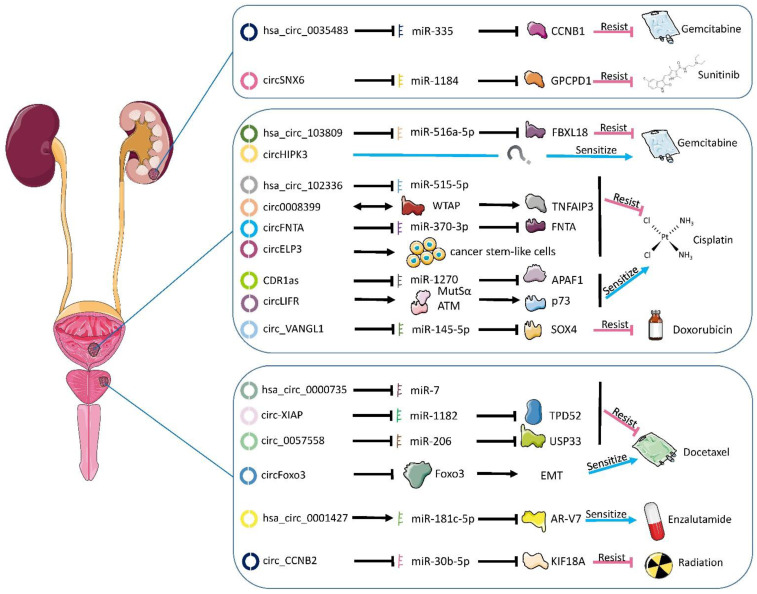
Functional circRNAs and their mechanisms in the development of chemotherapy and radiotherapy resistance in genitourinary. Mostly, circRNAs bind the miRNAs, thus preventing the latter from regulating their mRNA targets. Rarely, circRNAs directly interact with proteins to perform their functions. The blue/red arrow presents the effect on drug sensitivity if the circRNAs are overexpressed. Circ_CCNB2 and hsa_circ_0035483 are identical and share the same color. EMT: epithelial–mesenchymal transition.

Evidence suggested that circRNAs were involved in the onset and development of several diseases, such as cardiovascular [22], endocrinous [23], and neurological diseases [24]. They also play important roles in the progression of genitourinary cancers [25,26,27]. However, the exploration of circRNAs’ involvement in drug resistance of genitourinary cancers was limited. Here, we summarized the recent progress in the understanding of the functions and mechanisms of circRNAs in drug resistance of genitourinary cancers. A graphical illustration is provided in Figure 2.

## 2. CircRNAs and Drug Resistance in RCC

RCC is the most common type of kidney malignancy and the incidence of RCC is increasing, with an incidence of 4.4 per 100,000 for both sexes [27,28,29,30]. Most patients with RCC have local tumors without distant metastasis, the gold standard of treatment for this subset of patients is nephrectomy [31]. In contrast, there are still some patients who have had tumor metastasis at the time of diagnosis or recurrence after nephrectomy and systemic treatment should be applied for them [32]. Since renal cancers are not sensitive to radiotherapy and chemotherapy, targeted therapy and immunotherapy have been developed [33,34]. The initial first-generation immunotherapy targeting cytokines presented poor outcomes, while the tyrosine kinase inhibitors (TKI) greatly improved the prognosis and became the first-line therapy for advanced and metastatic RCC [35].

### 2.1. Gemcitabine Resistance in RCC

Gemcitabine, an analog of cytosine arabinoside, is an anti-tumor drug combating a wide range of diseases [36]. Gemcitabine has been used as a combination chemotherapy regimen for pancreatic cancer [37], BCa [38], non-small cell lung cancer (NSCLC) [39], biliary tract cancer [40], and cervical cancer [41]. As a single agent, gemcitabine could give an effective response rate of up to 31% in advanced RCC [42]. About a quarter of sarcomatoid and poor-risk RCC patients responded to the combination of gemcitabine and sunitinib [43]. Unfortunately, gemcitabineresistance occurs after use for a period of time [44,45].

Yan et al. found that upregulation of hsa_circ_0035483 promotes the drug resistance of renal cancer cells to gemcitabine, mainly hsa_circ_0035483 sponging hsa-miR-335, resulting in upregulated expression of Cyclin B1 (CCNB1) and thus promoting tumor growth and autophagy induced by gemcitabine in renal cancer cells [46]. Moreover, Cai et al. demonstrated that knockdown of Circ_CCNB2 (circBase ID: hsa_circ_0035483) can increase the radiosensitivity of PCa through repressing autophagy by the miR-30b-5p/Kinesin Family Member 18A (KIF18A) axis [47].

### 2.2. Sunitinib Resistance in RCC

Sunitinib, with strong anti-angiogenesis and direct anti-tumor activity, is a multi-target TKI. Its anti-tumor effect is mainly competitive with Vascular Endothelial Growth Factor (VEGF) to bind tyrosine kinase receptors, thus reducing angiogenesis—as angiogenesis is necessary for tumor growth [48]. Renal cell carcinoma is a highly vascularized tumor. The inactivation of the *Von Hippel–Lindau Tumor Suppressor (VHL)* gene and the activation of Hypoxia-Inducible Factor (HIF) promote the production of VEGF, while sunitinib blocks this process and plays an anti-tumor role [49]. Benefitting from the sunitinib, the overall survival of metastatic RCC has improved from 13 months in the cytokine era to 30 months [50]. For favorable-risk metastatic RCC patients, sunitinib is still the first-line systemic therapy [2].

Huang et al. found that the expression of cicrSNX6 (hsa_circ_0031607) was up-regulated in sunitinib-resistant renal cancer cell lines by RNA-seq, and verified that circSNX6 can sponge miR-1184 and inhibit Glycerophosphocholine Phosphodiesterase 1 (GPCPD1), and then increased the content of Lysophosphatidic Acid (LPA) in cells, which eventually led to the enhancement of drug resistance of renal cancer cells to sunitinib [51]. The expression of circSNX6 was also significantly upregulated in hepatocellular carcinoma and its function in hepatocellular carcinoma has not been determined [52].

## 3. CircRNAs and Drug Resistance in BCa

BCa ranks fourth among male malignant tumors and is more common in female malignancies. According to statistics, there are about 81,400 new cases and 17,980 deaths from BCa in the United States in 2020 [1]. Based on the depth of BCa invasion, BCa could be divided into non-muscular invasive BCa (NMIBC) and muscular invasive BCa (MIBC) and more than two-thirds of BCa belong to NMIBC [53]. The main treatment of NMIBC is transurethral resection of the bladder tumor plus intravesical chemotherapy, such as gemcitabine and doxorubicin, while MIBC patients are usually treated with radical cystectomy and neoadjuvant chemotherapy [54]. Neoadjuvant chemotherapy has also evolved over the past few decades. Methotrexate, vinblastine, doxorubicin, and cisplatin (MVAC) were used in the treatment of BCa since 1985, but, in recent years, patients with gemcitabine plus cisplatin (GC) neoadjuvant chemotherapy showed better tolerance than the former [55]. The new combination of cisplatin adjuvant chemotherapy improves the overall survival of patients. Combination chemotherapy based on cisplatin is currently the first-line therapy option for advanced and metastatic BCa [4].

### 3.1. Gemcitabine and Doxorubicin Resistance in BCa

Gemcitabine plus cisplatin has become one of the neoadjuvant chemotherapy regimens before radical cystectomy and has improved the survival rate of patients with BCa [56]. Gemcitabine is also used for some moderate-risk superficial NMIBC [57]. Huang et al. found that the high expression of circRNA hsa_circ_103809 in BCa increased tumor resistance to gemcitabine. Hsa_circ_103809 acts as the sponge of miR-516a-5p, resulting in the upregulation of F-box and Leucine-rich Repeat Protein 18 (FBXL18) expression, thus promoting tumor drug resistance [58]. The expression of hsa_circ_103809 is also increased in gastric cancer [59] and breast cancer [60], which regulates cell migration and invasion by binding to miR-101-3p and PI3K/Akt, respectively. However, in hepatocellular carcinoma and colorectal cancer (CRC), the expression of hsa_circ_103809 is downregulated and its overexpression can inhibit the progression of cancer cells [61,62].

Unlike the elevated expression of hsa_circ_103809 in BCa, circHIPK3 (hsa_circ_0000284) is downregulated in BCa and its expression can promote the sensitivity to gemcitabine [63]. The expression of circHIPK3 also negatively correlates with pathological grade and lymph node metastatic status. Moreover, circHIPK3 expression could serve as an independent prognostic biomarker for disease-free survival of BCa patients. In other tumors, circHIPK3 can also manipulate drug sensitivity. In pancreatic cancer, circHIPK3 sponging miR-330-5p promotes the expression of the Ras-associated Domain Family 1 (RASSF1), which leads to resistance to gemcitabine [64]. In addition, circHIPK3 also regulates the drug resistance of breast cancer cells to paclitaxel through the circHIPK3/miR-1286/Hexokinase 2 (HK2) axis [65]. Circ-HIPK3 is highly expressed in temozolomide-resistant glioma cells and promotes tumor growth and drug resistance [66]. At the same time, circHIPK3 can also regulate the resistance of CRC to oxaliplatin through the autophagy pathway [67].

Circ_VANGL1 (hsa_circ_0002623) can promote the growth of BCa and doxorubicin resistance, the mechanism is that circ_VANGL1 reduces miR-145 and promotes the expression of SRY-box Transcription Factor 4 (SOX4), and knockout of circ_VANGL1 can have the opposite effect [68]. In addition to the above axis, circ_VANGL1 can also sponge miR-605-3p, promote the expression of VANGL Planar Cell Polarity Protein 1 (VANGL1), and facilitate the progression of BCa [69]. Circ_VANGL1 also regulates the progression of non-tumor diseases, and its expression is downregulated in osteoporosis, when compared with non-osteoporosis patients. Mechanically, Circ_VANGL1 can absorb miRNA-217, which can target the 3’-UTR region of RUNX Family Transcription Factor 2 (RUNX2), thus promoting osteogenic differentiation [70].

### 3.2. Cisplatin Resistance in BCa

Cisplatin is a recognized antineoplastic drug, and its mechanism is generally believed to be that it binds to genomic DNA and then interferes with normal transcription or DNA replication, resulting in cancer cell death [71]. For advanced and metastatic BCa, cisplatin-based chemotherapy is currently the first-line therapy for advanced and metastatic BCa [4]. For MIBC, neoadjuvant cisplatin-based chemotherapy might bring a downgrade of disease and improved prognosis [72,73]. A meta-analysis of adjuvant treatment trials has shown a 25% reduction in risk of death with cisplatin-based adjuvant treatment [74]. However, almost half of the patients would experience progression under the GC or dose-dense MVAC regimen within 3 years [75]. Cisplatin resistance prevents these patients from having satisfying outcomes.

Circ_0008399 (hsa_circ_0008399), a newly found circRNA in BCa, binds to the Wilms’ Tumor 1-Associating Protein (WATP) and promotes the expression of TNF α-inducible Protein 3 (TNFAIP3) by increasing its mRNA stability in an m^6^A-dependent manner, resulting in cisplatin resistance [76]. TNFAIP3 is described as an anti-apoptotic protein that inhibits TNF-induced apoptosis, and knockdown of it could also promote apoptosis in cisplatin-resistant BCa cells upon cisplatin treatment [77]. Su et al. verified that CircELP3 (hsa_circ_0001785) may promote cisplatin resistance in BCa by targeting tumor stem-like cells [78]. Not only that, but hsa_circ_102336 can also promote cisplatin resistance through the sponging of miR-515-5p [79], and another circRNA, circFNTA (hsa_circ_0084171), has a similar function by regulating the miR-370-3p/Farnesyltransferase Subunit Alpha (FNTA)/KRAS axis [80]. In addition, miRNA-451a is downregulated by circ-FNTA, and the effect of miRNA-451a is to reduce the level of Sphingosine-1-phosphate Receptor 3 (S1PR3). The increase of circ-FNTA promotes the progression and chemo-resistance of BCa [81].

Conversely, circLIFR (hsa_circ_0072309) augmented the interaction between MutSα and ATM, ultimately contributing to stabilizing the p73 protein level [82]. Vascular smooth muscle (VSMCs) dysfunction is the key to the occurrence and development of intracranial aneurysms. CircLIFR regulates the function of VSMCs through the miR-1299/Kinase Insert Domain Receptor (KDR) axis, including migration, invasion, and apoptosis [83].

Cerebellar Degeneration-associated Protein 1 Antisense RNA (CDR1as, also known as ciRS-7, circBase ID: hsa_circ_0001946), as one of the most widespread circRNA, plays an important role in the occurrence and development of many kinds of tumors [84]. CDR1as regulates the resistance of BCa to cisplatin by regulating the miR-1270/Apoptotic Protease-Activating Factor 1 (APAF1) axis [85,86]. CDR1as also promotes the resistance of NSCLC to cisplatin through miR-641/Homeobox Protein Hox-A9 (HOXA9) pathway [87], while in ovarian cancer it shows the opposite effect through the miR-1270/Suppressor of Cancer Cell Invasion (SCAI) signal pathway [88]. In addition to cisplatin, CDR1as contributes to drug resistance to other chemotherapies. In lung adenocarcinoma cells, the expression of CDR1as is positively correlated with resistance to pemetrexed and cisplatin, and the mechanism of drug resistance is mainly through the EGFR/PI3K signal pathway [89]. Yang et al. verified that CDR1as is upregulated in 5-fluorouracil (5-FU)-resistant breast cancer cells. It can bind to miR-7 to further regulate Cyclin E1 (CCNE1). Inhibition of CDR1as can increase the chemosensitivity of breast cancer cells to 5-FU [90].

Chi et al. measured the expression of hsa_circ_0000285 by quantitative PCR and found that it was significantly reduced in BCa tissues and serum compared to adjacent tissues and healthy controls [91]. It was further lowered in cisplatin-resistant BCa patients than cisplatin-sensitive and it can be an independent prognostic factor for the outcomes of BCa patients [91].

## 4. CircRNAs and Drug Resistance in PCa

Localized PCa could be curatively managed by RP or RT. Unfortunately, about 20–40% of post-RP patients [92,93] and 30–50% of post-RT patients [94] will experience biochemical recurrence within 10 years. Meanwhile, approximately 4% of PCa patients have metastatic disease at the time of diagnosis in western countries [95], and the incidence would be much higher in China [96]. For relapsing and de novo metastatic patients, androgen deprivation therapy (ADT) is the recommended therapy, and it could significantly reduce serum testosterone and prostate-specific antigen (PSA) levels [97]. However, most patients would progress from a hormone-sensitive state (HSPC) to castration-resistant PCa (CRPC) within 2–3 years [98]. The taxanes, such as docetaxel and novel androgen receptor signaling inhibitors (ARSi) including abiraterone, enzalutamide, and apalutamide, could extend the survival of CRPC [99,100], and this indication was further expanded to high-risk metastatic HSPC patients [101,102,103,104].

### 4.1. ADT Resistance in PCa

The transition from HSPC to CRPC is the critical milestone of PCa development and the two stages present different genomic landscapes [105,106]. Using a high throughput micro-array assay on a panel of prostate cell lines, Greene et al. identified several circRNAs that are associated with androgen independence [107]. Luo et al. found an *androgen receptor (AR)* circular RNA, called circAR3, that is widely expressed in PCa and reduced when tumors progressed to CRPC [108]. Interestingly, circAR3 correlates with clinical parameters such as Gleasons score and treatments but does not affect AR signaling, PCa cell proliferation, and invasion. Furthermore, Cao et al. identified 13 upregulated circRNAs generated from the *AR* in CRPC, and their expression correlated strongly with that of the linear *AR* transcripts [109]. These studies revealed the different circRNA profiles in CRPC and HSPC, but the roles of these circRNAs in the progression to CRPC need further investigation.

### 4.2. Docetaxel Resistance in PCa

Docetaxel has been applied to CRPC since the 1990s and the mechanism behind its resistance is the most well-studied [110]. Hsa_circ_0000735 was upregulated in docetaxel-resistant PCa and knockdown of it boosted docetaxel sensitivity, constrained viability and progression, and fostered apoptosis of docetaxel-resistant PCa cells [111]. Mechanistically, miR-7 is sponged by hsa_circ_0000735, and downstream targets including Multidrug Resistance Protein 1 (MDR1), CyclinD1, and B Cell Lymphoma 2 (BCL-2) were reduced after hsa_circ_0000735 interference. Additionally, hsa_circ_0000735 was found to be involved in docetaxel-resistance in ovarian cancer via the hsa_circ_0000735/miR-546b/Dickkopf-related protein 4 (DKK4)/P-glycoprotein (p-GP) axis [112]. It can also enhance proliferation, migration, invasion, and glycolysis of NSCLC cells by targeting the miR-940/Bone Morphogenetic Protein Binding Endothelial Cell Precursor-Derived Regulator (BMPER) axis [113].

Similarly, circ_0057558 (has_ circ_0057558) could sponge miR-206, thereby regulating the Ubiquitin Specific Peptidase 33 (USP33)/c-Myc axis in PCa. Knockdown of it repressed cell proliferation and colony formation, and resulted in docetaxel resistance [114]. It was also reported that circ_0057558 promotes non-alcoholic fatty liver disease by regulating miR-206/Rho-kinase 1 (ROCK1)/AMPK signaling [115].

Circ-XIAP (has_circ_0005276) was upregulated in not only docetaxel-resistant PCa tissue specimens and cell lines but also exosomes from docetaxel-resistant cells [116]. Experiments validated that it promotes docetaxel resistance of PCa by regulating the miR-1182/Tumor Protein D52 (TPD52) axis. Alternatively, it can interact with FUS binding protein (FUS) so as to activate the transcription of the X-linked Inhibitor of Apoptosis Protein (XIAP), thereby promoting cell proliferation, migration, and epithelial–mesenchymal transition (EMT) [117].

In contrast to these resistance-promoting circRNAs, circFoxo3 (has_circis_0006404) could repress Forkhead Box O3 (FOXO3) protein expression and EMT, leading to enhanced chemosensitivity to docetaxel [118]. CircFoxo3 is composed of the second exon of the *FOXO3* gene containing 1435 nucleotides and it displays high conservation levels between humans and mice [119]. It is also involved in the progression of cancers including glioblastoma [120], gastric carcinoma [121], oral and esophageal squamous cell carcinoma [122,123], and BCa [124,125]. Particularly, circFoxo3 expression was increased in adriamycin-resistant hepatocellular carcinoma tissues and cell lines, and its overexpression enhances hepatocellular carcinoma invasion and growth via the miR-199a-5p/ATP Binding Cassette Subfamily C Member 1 (ABCC1) axis [126]. Moreover, the role of circFoxo3 in cardiac and neurologic diseases has also been well-explored [127,128,129,130].

### 4.3. ARSi Resistance in PCa

Another fundamental therapy for PCa is the novel hormone therapy (NHT) that targets AR signaling. Compared with docetaxel chemotherapy, ARSi could offer non-inferior cancer control and improved quality of life [131]. However, primary and acquired resistance to these agents limited the population that could benefit from them. Many hypotheses about the mechanism have been proposed, including intratumoral androgen biosynthesis, neuroendocrine differentiation, and AR alterations [132]. AR-V7 is a splice variant of AR and its expression infers resistance to ADT and NHT [133,134,135]. Wu et al. found that suppressing circRNA17 (hsa_circ_0001427) in PCa cells could increase AR-V7 expression that might then lead to enzalutamide resistance [136]. Interestingly, circRNA17 functions as a reservoir, not a sponge, to increase the expression of miR-181c-5p, which could directly bind to the 3’UTR of AR-V7. Wu et al. also verified that adding circRNA17 or miRNA-181c-5p could suppress the growth of the enzalutamide-resistant cells using the in vivo mouse model [136].

Several other studies also explored the circRNA profiles of enzalutamide-resistant PCa. Greene et al. screened the circRNA expression of two enzalutamide-resistant LNCaP clones against the control cell line and found 111 commonly altered circRNAs [137]. Yu et al. further analyzedGreene et al.’s data and constructed a ceRNA regulatory network [138]. They chose hsa_circ_0047641 and further validated its effect on the proliferation of enzalutamide-sensitive and resistant PCa cells. Xiang et al. detected low expression of circUCK2 (hsa_circ_001357) in enzalutamide-resistant cells, and knocking down circUCK2 increased cell invasion and proliferation via the miR-767-5p/Tet Methylcytosine Dioxygenase 1 (TET1) axis [139]. CircUCK2 is also essential in ischemic stroke, and overexpression of it improved neurological deficits via the circUCK2/miR-125b-5p/Growth Differentiation Factor 11 (GDF11) axis [140].

## 5. Mechanisms

**Figure 3:**
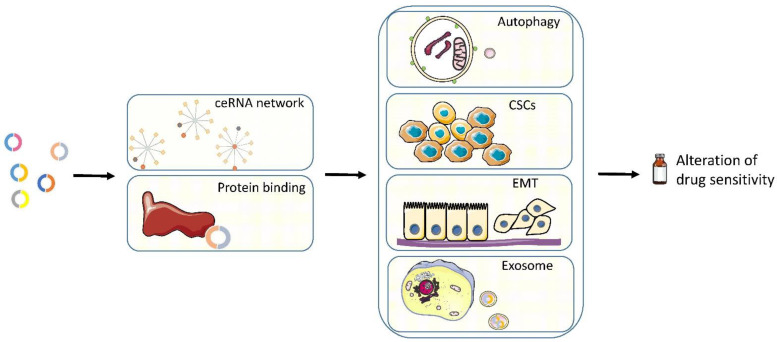
The mechanisms via which circRNAs regulate the sensitivity to systematic treatments in genitourinary cancers. CSCs: cancer stem cells, EMT: epithelial–mesenchymal transition.

The studies described above thoroughly explored the function of circRNAs in drug resistance. The mechanisms of circRNAs are quite varied and some circRNAs could function via a combination of several mechanisms. In Figure 3, we listed the most recognized mechanisms in circRNA-related drug resistance.

### 5.1. CeRNA Network

The ceRNA network is a typical example of the post-transcription regulation of gene expression. Transcripts can regulate each other by competing for shared miRNAs. The ceRNA network comprises protein-coding mRNAs as well as non-coding RNAs, including long non-coding RNAs (lncRNAs), circRNAs, and pseudogene RNAs. Via the ceRNA network, non-coding RNAs could contribute to anti-cancer drug resistance [141]. In recent years, the studies on the mechanism of action of circRNA in drug resistance are mostly based on the ceRNA network. As shown in Figure 2, the ceRNA network is still the main approach to drug resistance in genitourinary cancers.

Both circRNAs and mRNAs contain the binding sites (also termed miRNA response elements (MREs)) for miRNAs, and they can competitively bind to the shared miRNA. In general, circRNAs could bind to miRNAs, consequently interrupting the binding between mRNAs and miRNAs and affecting the downstream mRNAs’ levels. The binding affinity of miRNA and its targets is mainly influenced by the matching between MREs and the miRNA seed regions [142]. Intuitively, RNA base alterations including single-nucleotide polymorphism (SNP) and RNA editing could affect the binding affinity and even change the target spectrum [143,144]. Also, RBPs could regulate the ceRNA network. RBPs could occupy the MREs and hamper the miRNA-target binding [145,146]. On the other hand, RBPs could promote miRNA-target binding via recruiting miRNAs to the target [147].

Mostly, circRNAs function as a sponge of miRNAs, but, in some cases, circRNAs could also be a miRNA reservoir. In the regulatory network of ciRS-7, miR-7, and miR-671, miR-671-directed cleavage of ciRS-7 could release miR-7 and result in prompt and efficient repression of miR-7 targets [148]. Similarly, circ-HIAT1 (has_circ_001013) protects three of its targets from the inhibition of the AR and suppresses the downstream Cell Division Cycle 42 (CDC42) level [149]. In enzalutamide-resistant PCa, circRNA17 functions as a reservoir to increase the expression of miR-181c-5p and decrease the expression of AR-V7, which is a fundamental driver for ARSi-resistance [136].

### 5.2. Binding Proteins

Besides sponging miRNAs, circRNAs could also directly bind to proteins to perform their functions. This mechanism has also been investigated in the development of drug resistance. In acute myeloid leukemia (AML), the circMYBL2 (hsa_circ_0006332) could directly bind to Polypyrimidine Tract Binding Protein 1 (PTBP1), leading to AML progression and quizartinib resistance [150]. In breast cancer, circMTO1 (hsa-circ-007874) interacts with TNF Receptor Associated Factor 4 (TRAF4), consequently inhibiting the mitotic kinesin Eg5 protein level and reversing monastrol resistance [151].

In BCa, hsa_circ_0008399 could bind to WATP and promote cisplatin resistance by targeting the circ0008399/WTAP/TNFAIP3 axis [76]. Also, circLIFR can interact with MutS Homolog 2 (MSH2) and augment the interaction between MutSα (formed by MSH2 and MSH6) and ATM [82], which attenuated BCa cisplatin resistance.

### 5.3. Autophagy

Autophagy is a process in which the cell self-digests its own components through the delivery of cytoplasmic cargo to the lysosome [152]. Autophagy could be induced by a wide range of cancer therapies. The mechanism of autophagy in cancer chemoresistance has been investigated by many studies and it has been shown to promote cancer cell survival and drug resistance [153]. Additionally, autophagy inhibition could sensitize the tumor cells to therapies, which makes autophagy a promising therapeutic target [154,155].

In the mechanism of circRNAs in drug resistance, the crucial involvement of autophagy has been identified in several cancers. In NSCLC, high hsa_circ_0085131 levels activated the hsa_circ_0085131/miR-654-5p/Autophagy Related 7 (ATG7) axis and cell autophagy to enhance cancer cell cisplatin resistance [156]. The circMTO1 sponges miR-6893 to promote S100 Calcium Binding Protein A1 (S100A1) expression and eventually contributes to chemoresistance enhancement in cervical cancer, while autophagy inhibitor 3-Methyladenine could reverse the effect [157]. In papillary thyroid carcinoma, circEIF6 (hsa_circ_0060060) could promote autophagy induced by cisplatin, enhancing the cisplatin resistance [158]. In AML and chronic myeloid leukemia, circPAN3 (hsa_circ_0100181) and hsa_circ_0009910 could induce autophagy to augment resistance to doxorubicin and imatinib, respectively [159,160].

In RCC, hsa_circ_0035483 could facilitate gemcitabine-induced autophagy and enhance the resistance of RCC to gemcitabine by regulating the hsa-miR-335/CCNB1 axis [46]. In PCa, knockdown of circ_CCNB2 (hsa_circ_0035483) can amplify radiosensitivity through inhibiting autophagy by targeting the miR-30b-5p/KIF18A axis [47].

### 5.4. Cancer Stem Cell (CSC)

Cancer stem cells constitute subpopulations of cancer cells with an increased renewal capacity and the ability to recapitulate the heterogeneity in primary tumors [161]. Dormant CSCs are capable of escaping from harmful stress, such as radiotherapy and chemotherapy.

CircRNAs such as hsa_circ_001680 and circ-NOTCH1 (hsa_circ_0089547) have been found to promote CSC populations in cancer [162,163]. More importantly, hsa_circ_001680 could enhance stemness in CRC and induce irinotecan therapeutic resistance [162]. In BCa, lower circELP3-expressing cancer cells displayed lower sphere formation ability and stem cell marker expression, and repressed progression and drug resistance [78].

### 5.5. EMT

EMT is a transdifferentiation process driven by transcriptional regulations and epigenetic modifications, through which the cells lose epithelial characteristics and transit to mesenchymal phenotypes. The role of EMT in tumor drug resistance has been gradually recognized and explored, and repression of EMT could render cancer cells more chemosensitive [164].

The circBMPR2 (hsa_circRNA_0003218) could relieve the tamoxifen resistance of breast cancer [165]. During the process, western blot showed that knockdown of circBMPR2 could decrease the levels of epithelial markers and increase the levels of mesenchymal markers. In breast cancer, circKDM4C (has_circ_0001839) suppresses tumor EMT and attenuates doxorubicin resistance by sponging miR-548p [166]. Circular RNA cESRP1 (hsa_circ_0084927) binds to miR-93-5p to regulate Transforming Growth Factor-β (TGF-β)-mediated EMT in small cell lung cancer and silencing it causes chemoresistance [167]. Similarly, circRNAs were found to alter drug sensitivity by affecting EMT in other cancers, including lung adenocarcinoma [168], nasopharyngeal carcinoma [169], and esophageal cancer [170,171]. In PCa, the circFoxo3 could repress FOXO3 level and EMT, leading to cancer cell survival, migration, invasion, and chemoresistance to docetaxel [118].

### 5.6. Exosome

Exosomes are vesicles of endocytic origin and are important mediators of intercellular communication, containing mRNAs, miRNAs, lncRNAs, circRNAs, proteins, lipids, and transcription factors. The circRNAs are enriched and stable in exosomes, and exosomal circRNAs can be tumor biomarkers as well as therapeutic targets in cancers [172]. Moreover, exosomal circRNAs are involved in the development of drug resistance. For example, in small cell lung cancer, serum exosomal FLI1 exonic circular RNA 1 (FECR1) was associated with poor survival and clinical response to chemotherapy [173]. Hon et al. found that exosomes could transfer chemoresistance from FOLFOX (fluorouracil plus oxaliplatin)-resistant CRC cells to parental CRC cells, and hsa_circ_0000338 may play a pro-carcinogenic role in exosomes and enhance drug resistance of recipient cells [174]. Exosomal ciRS-122 (hsa_circ_0005963) could also induce oxaliplatin resistance in CRC cells, while exosomal transfer of circ-FBXW7 enhanced oxaliplatin sensitivity [175,176]. Similar functions of exosomal circRNAs have also been identified in NSCLC [177], glioma [178], hepatocellular carcinoma [179], multiple myeloma [180], epithelial ovarian cancer [181], osteosarcoma [182], and pancreatic cancer [183]. In PCa, the exosomal circ-XIAP could promote docetaxel resistance by regulating the miR-1182/TPD52 axis [116].

Furthermore, Chen et al. revealed that cancer cell-derived exosomal circUSP7 (hsa_circ_0005152) could induce CD8 + T cell dysfunction and anti-PD1 resistance in NSCLC [184]. Zhang et al. also found that cancer cell-derived exosomal circUHRF1 (hsa_circ_0048677) could cause resistance to anti-PD1 therapy in hepatocellular carcinoma by natural killer cell exhaustion [185]. These studies suggested that the exosomal circRNAs might alter the responsiveness of the immune checkpoint inhibition (ICI) therapies.

## 6. Limitations and Perspectives

Several mechanisms for circRNA-related drug resistance have been discovered and these studies were important for interpreting drug resistance. Nevertheless, some aspects need reinforcement to help us better understand the role of circRNAs in resistance development.

In the above-mentioned studies, investigators explored the circRNAs’ function in RCC, BCa, and PCa. However, little is known about the role of circRNAs in other genitourinary cancers, such as testicular cancer and penile cancer. Further studies on these cancers could provide more information about the circRNAs molecular mechanisms and clinical utility.

As listed in Table 1, several studies unveiled the different circRNA patterns in drug-resistant cancers. These works supported the association between circRNAs and the drug resistance status. They could serve as prognostic biomarkers in cancers [91,108]. Some studies, Luo et al. [108] and Yu et al. [138] for example, further illustrated the functions of these circRNAs in cancer invasion and proliferation. However, it should be noted that the effect of these circRNAs on drug resistance was not all experimentally validated, which is mandatory for ascertaining the causal–effect relationship. Future studies to confirm the functions of these differently expressed circRNAs are warranted.

As discussed above, several mechanisms behind circRNA-related drug resistance have been identified. The ceRNA network is the central route for resistance, and most studies focused on downstream molecular signaling and the consequent phenotype. Cellular and organelle mechanisms, including autophagy [46,47], cancer stem-like cell [78], exosome [116], and EMT [118], are also involved during drug resistance and they could be explored in future studies as possible resistance drivers and therapeutic targets.

Previous studies focused on chemotherapy (e.g., gemcitabine, cisplatin, and docetaxel) and ARSi resistance, and presented the contribution of circRNAs well, especially the cisplatin resistance of BCa. Due to insensitivity to chemotherapy and radiation, the drug resistance in RCC is relatively less studied. As a promising strategy, ICI therapies that block PD-1 or T-lymphocyte-associated antigen 4 (CTLA-4) signaling are being rapidly developed for cancer treatment and showed superior efficacy for intermediate- and poor-risk RCC [186]. A combination of ICI and anti-angiogenic therapy also displayed a better prognosis for RCC [187]. In BCa, pembrolizumab (PD-1 inhibitor) and atezolizumab (PD-L1 inhibitor) have been tested on cisplatin-ineligible patients in phase 2 trials [188,189]. They have been approved for patients who progressed during or after platinum-based combination chemotherapy and could also be considered as first-line treatment in patients unfit for platinum-based chemotherapy [4]. Unfortunately, except for some cancers that are sensitive to immunotherapy such as melanoma, the response rate to single-agent PD-1 blockade in most approved diseases is limited to 10–25% [190]. In other cancers such as NSCLC and hepatocellular carcinoma, circRNA function in ICI resistance has been explored [184,185,191,192]. ICI immunotherapy, or combination with other anticancer drugs, has shown promising efficacy in urological cancers, and are likely to reshape the therapeutic scenario of urological cancers [193]. Understanding the mechanisms of de novo and acquired resistance to ICI immunotherapy is an essential topic in the future and the circRNA’s role in it needs to be clarified.

Based on the double-edged role of circRNAs in drug resistance, different strategies could be applied to ameliorate drug responsiveness. For tumor-suppressive circRNAs, introducing exogenous purified or engineered circRNAs might lead to new and efficient approaches for cancer therapy [194,195]. The stability and long half-life of circRNAs allow a long-term anticancer effect. RNA interference specifically targeting oncogenic circRNAs can also effectively inhibit cancer development. Both the circRNA-targeting antisense oligonucleotide (ASO) and cholesterol-conjugated small interfering RNAs (siRNAs) injection could induce cancer repression in patient-derived xenograft models [196,197,198]. These preclinical investigations confirmed circRNAs aspromising therapeutic targets, and their clinical utility warrants further validations.

## 7. Conclusions

In this review, we summarized the recent investigations on the role of circRNAs in drug resistance of genitourinary cancers. The circRNA profiles differed after drug resistance and the mis-regulation of circRNA could greatly contribute to cancer progression and drug resistance. These findings emphasized the essential participation of circRNAs and provided a better understanding of drug resistance. Further studies should focus on the resistance to updated therapies, such as immunotherapy, as well as verifying circRNAs as promising therapeutic targets.

## Figures and Tables

**Table 1:** Summary of studies that investigated the circRNAs profile after drug resistance.

Study	Cancer	Comparison	Differently Expressed circRNA	Expression Alteration	Further Investigation
[91]	Bladder cancer	T vs. N and cisplatin-resistant vs. naive	hsa_circ_0000285	↓	Prognosis
[108]	Prostate cancer	CRPC vs. HSPC	circAR3	↓	Invasion, proliferation, downstream pathways
[109]	Prostate cancer	CRPC vs. HSPC	13 circARs	↑	Association with linear AR transcript
[137]	Prostate cancer	EnzR vs. naive	111 circRNAs	NA	NA
[139]	Prostate cancer	EnzR vs. naive	circUCK2	↓	Invasion, proliferation
[138]	Prostate cancer	EnzR vs. naive	4 circRNAs	↑	
			9 circRNAs	↓	
			hsa_circ_0047641	↑	Invasion, proliferation

T = tumor, N = normal, CRPC = castration-resistant prostate cancer, HSPC = hormone sensitive prostate cancer, EnzR = enzalutamide-resistant, NA = not available. The arrows present if the expression of circRNAs elevated (↑) or decreased (↓) in drug-resistant cancers.

## Data Availability

Not applicable.
